# Transcatheter Tricuspid Valve Therapy: From Anatomy to Intervention

**DOI:** 10.3389/fcvm.2021.778445

**Published:** 2021-11-18

**Authors:** Valeria Cammalleri, Myriam Carpenito, Maria Caterina Bono, Simona Mega, Gian Paolo Ussia, Francesco Grigioni

**Affiliations:** Department of Cardiology, Policlinico Universitario Campus Bio-Medico, Rome, Italy

**Keywords:** tricuspid valve, tricuspid anatomy, echocardiography, computed tomography, transcatheter tricuspid valve intervention

## Abstract

Nowadays, severe symptomatic tricuspid regurgitation (TR) affects millions of persons worldwide. However, the benefit of surgical correction of isolated secondary TR remains controversial because of the increased risk of periprocedural mortality and morbidity. In recent years, novel transcatheter tricuspid valve interventions (TTVI) were developed to treat TR, so that TTVI is currently considered in symptomatic, inoperable, anatomically eligible patients. TTVI can be divided into these five domains: edge-to-edge leaflet repair, tricuspid annuloplasty, caval implants, spacer, and total valve replacement. Each transcatheter intervention needs specific imaging protocols for assessing the anatomical feasibility and consequentially predicting the procedural success. This review summarizes the available multimodality imaging tools for screening patients with TR, and identifies anatomical characteristics to choose the best option for the patient.

## Introduction

Interest in the tricuspid valve (TV) has increased over the past two decades, because of the recognition of the progressive nature and clinical impact of secondary tricuspid regurgitation (TR) on outcomes (1, 2). Furthermore, the high in-hospital mortality associated with isolated TV surgery complicates the management of this valvular heart disease, especially in elderly and frail patients who often suffer from multiple comorbidities (1–4).

In recent years, novel transcatheter tricuspid valve interventions (TTVI) were developed for treating TR, so that the current european society of cardiology (ESC) and the european association for cardio-thoracic surgery (EACTS) guidelines for the management of valvular heart disease consider TTVI in symptomatic and inoperable patients who met anatomical criteria for eligibility (5). Numerous transcatheter devices are currently under clinical investigation or being developed. Most procedures mimic the left atrioventricular valve interventions and follow similar underlying principles: leaflet repair improving coaptation, annuloplasty reducing annular size, and implant of a prosthesis in heterotopic or orthotopic position. However, a complete knowledge of the anatomy of the TV complex is crucial for understanding the process through which secondary TR occurs and realizing the role of transcatheter interventions. Each transcatheter intervention needs specific imaging protocols to state the anatomical feasibility and subsequently predict the procedural success.

This article reviews the TV anatomy and the available imaging tools for a comprehensive assessment of the valve. Finally, it identifies favorable and unfavorable anatomical conditions that can guide the operator to choose the best option for the patient.

## Anatomy of the TV Complex

The TV is the largest valve with a normal orifice area of 7–9 cm^2^ and the most apically located valve. Its functional anatomy consists of four elements named annulus, leaflets, papillary muscles, and chords (6–8). From the anatomical point of view, the TV annulus is a virtual structure connecting the right atrium (RA) and right ventricle (RV), in which a continuous ring of dense connective tissue does not exist. Three valve leaflets are present, named septal, posterior, and anterior leaflet, separated by three commissures, designed as Anteroseptal (AS), Post-eroseptal (PS), and Anteroposterior (AP). Generally, valve leaflets attach to the atrioventricular junction, but the AS half of the septal leaflet is on the ventricular side of the atrioventricular junction (8). The septal leaflet is the smallest one, arises directly from the tricuspid annulus above the interventricular septum, and it is connected to the septal wall through short chords.

For this reason, it is the least mobile of the three leaflets. The anterior leaflet is the largest one, whereas the posterior leaflet is characterized by the presence of multiple scallops (8). Modern anatomy textbooks and published reports suggest that the number of leaflets may vary. However, in our opinion, these observations arise because there is a lack of a clear definition of the commissures and the extent of the leaflets (9). Anatomically, the commissures have peculiar findings: they are supported by fan-shaped chords and do not open directly into the annulus, but a few millimeters of the leaflet tissue remain, similar to small scallops.

Therefore, at first, the commissures must be defined, then all tissues between them are part of the leaflets. Thus, the TV consists of a single septal leaflet, a large anterior leaflet, and a posterior leaflet, which can have a variable number of scallops. In some cases, the AP commissure can be indistinct, looking like a bicuspid valve.

All leaflets received chords from the related papillary muscles. Specifically, the anterior papillary muscle gives chords to the anterior and posterior leaflets, and the medial papillary muscle gives chords to the posterior and septal leaflets (8). The septal wall provides chords to the anterior and septal leaflets (8). In addition, accessorial chords arising for the right ventricular free wall and the moderator band can directly attach the leaflets. The pattern of chordal insertion in the TV is variable and consist of rough zone chords, free edge chords, deep chords, and basal chords (which are common to all three leaflets), and fan-shaped chords (which peculiarly insert into commissures or clefts of the posterior leaflet) (8).

## Geometric Features of the TV Complex

Three-dimensional (3D) reconstruction of the tricuspid annulus showed a non-planar saddle-shaped pattern with two high points, corresponding to the AS and post-erolateral segments, and two low points corresponding to the anterolateral and PS segments (10). The annular shape changes during the cardiac cycle being circular during diastole and ovoid during the systole. Consequently, the annular size is maximum in late diastole and minimum in mid-systole.

In patients with functional TR, the tricuspid annulus is flatter, which reduces the saddle shape. Compared with normal subjects, patients with functional TR have a more circular annulus, and greater enlargement in the AP than in the mediolateral distance, because the greater dilation occurs along the free-wall side of the annulus. In presence of TR, the percent reduction of the tricuspid annulus size during the systole is also significantly decreased. Therefore, the minimum systolic annular dimension is almost twice larger than in normal subjects (11).

## Multimodality Imaging in Patients With TR

Characterization of the anatomy of the RV and TV is the first step in the evaluation of patients with functional TR, candidates for TTVI. While historically the anatomical knowledge of TV was derived from cadaveric specimens or open, today non-invasive imaging techniques represent helpful instruments to understand the anatomy of cardiac structures. Currently, echocardiography, CT, and cardiac magnetic resonance (CMR) are available instruments to study the TV anatomy, guide in the decision-making process, and support the development of novel transcatheter therapies. In addition, the current ESC/EACTS guidelines for the management of valvular heart disease recommend transesophageal echocardiography (TEE) and cardiac CT for detailed anatomical evaluation owing to higher spatial resolution (5).

Echocardiography is undoubtedly the first step in assessing the etiology and severity of TR and the size and function of the RVs. Moreover, this is the only imaging technique available for intraprocedural TTVI guiding. But the anterior location can pose challenges for the transesophageal approach. CMR is the gold standard for assessing right ventricular size and function and can help in quantifying TR when echocardiographic doubt exists. However, the reduced availability and contraindications limit the routine application of CMR in this setting of patients. On the other hand, CT provides complete morphologic imaging of the heart structures, thanks to a high spatial resolution with excellent capacity to define the endocardial border. However, disadvantages are the need for radiation and iodinated contrast injection.

## The Role of Echocardiography

Transthoracic echocardiography (TTE) is the first-line approach for studying the anatomy and function of TV and right-side chambers (12). Due to the complex structure of the TV, and the difficulty in visualizing all three leaflets in a single two-dimensional (2D) plane, a comprehensive assessment using multiple windows is required (13). The following views are needed: parasternal RV inflow and short-axis, standard apical four-chamber, apical RV-focused four-chamber, subcostal four-chamber, and short-axis. However, identification of the tricuspid leaflets and other valve components from the standard transthoracic views remains challenging, because of the intrinsic anatomic structure of the TV, as well as the unpredictability of imaging planes that may be acquired with different angles of the transducer (6).

**Figure 1:**
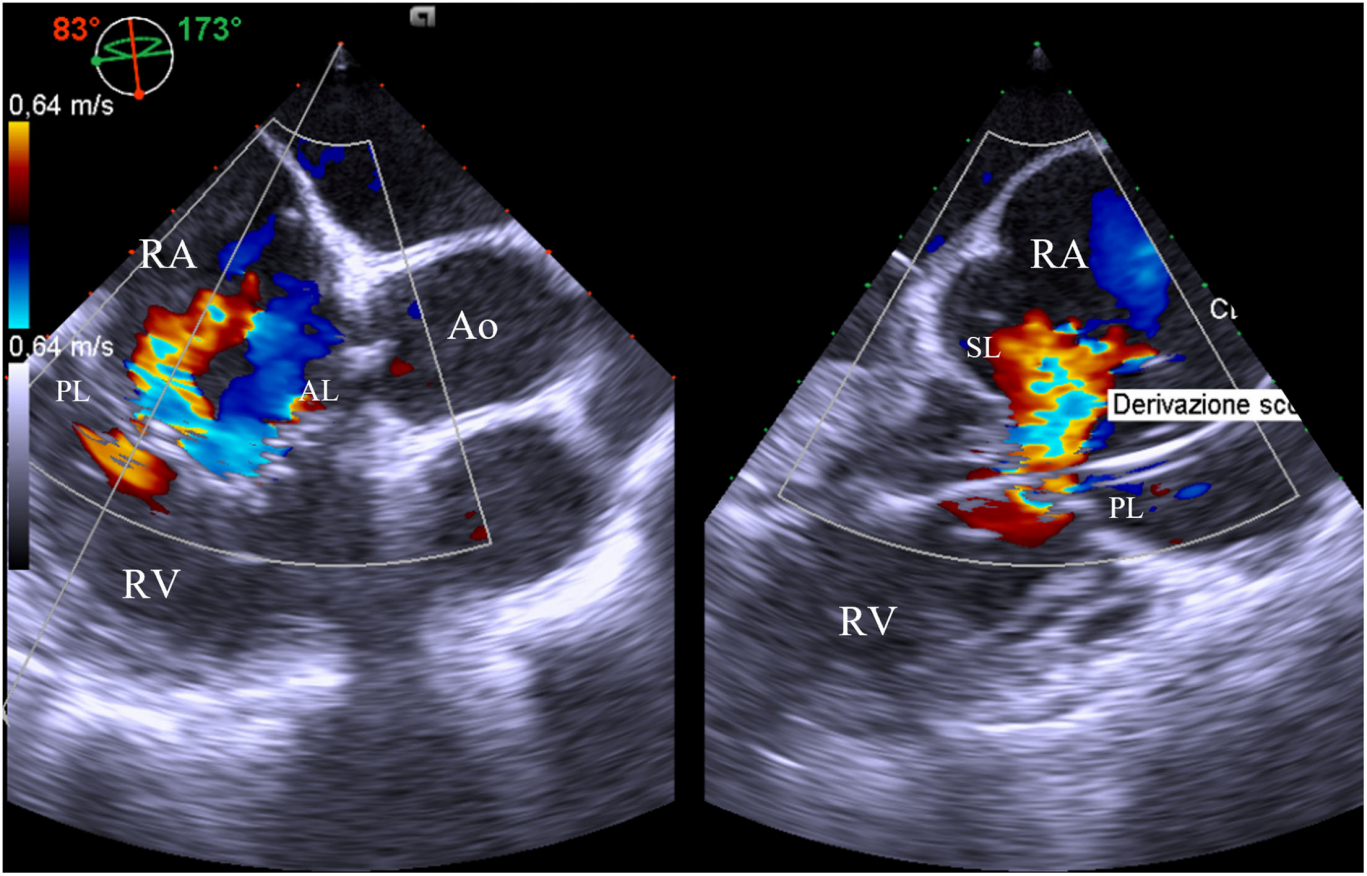
Mid-esophageal right ventricle inflow/outflow view with orthogonal multiplane, which crosses the coaptation between the septal and the posterior leaflet. At this level, it is identified the major origin of the jet, as well as the presence of pacemaker lead interfering with mobility of the posterior leaflet. AL, anterior leaflet; Ao, aorta; PL, posterior leaflet; RA, right atrium; RV, right ventricle; SL, septal leaflet.

Transesophageal echocardiography is the second-line imaging modality for the assessment of TV anatomy in patients with TR, even if the anterior position of the valve can limit the examination. Nevertheless, TEE allows obtaining imaging from several depths (mid-esophageal, distal-esophageal, shallow transgastric, and deep transgastric views) and multiplane angles. The four-chamber view (0–0°) and the alternative four-chamber view (160–180°) permit the visualization of the septal and typically the anterior leaflet; the posterior leaflet can also be visualized by changing probe angulation. Simultaneous biplane imaging may clarify which leaflet is imaged because the anterior leaflet is usually adjacent to the aorta, whereas the coronary sinus is the marker for the posterior leaflet. Other useful information for a comprehensive TV assessment may be achieved by a mid/deep esophageal RV inflow/outflow view in multiplane (60–100°), which crosses the coaptation between the septal and the posterior leaflet (near the lateral annulus) and the septal and anterior leaflet (near the aorta) (14) (Figure 1).

**Figure 2:**
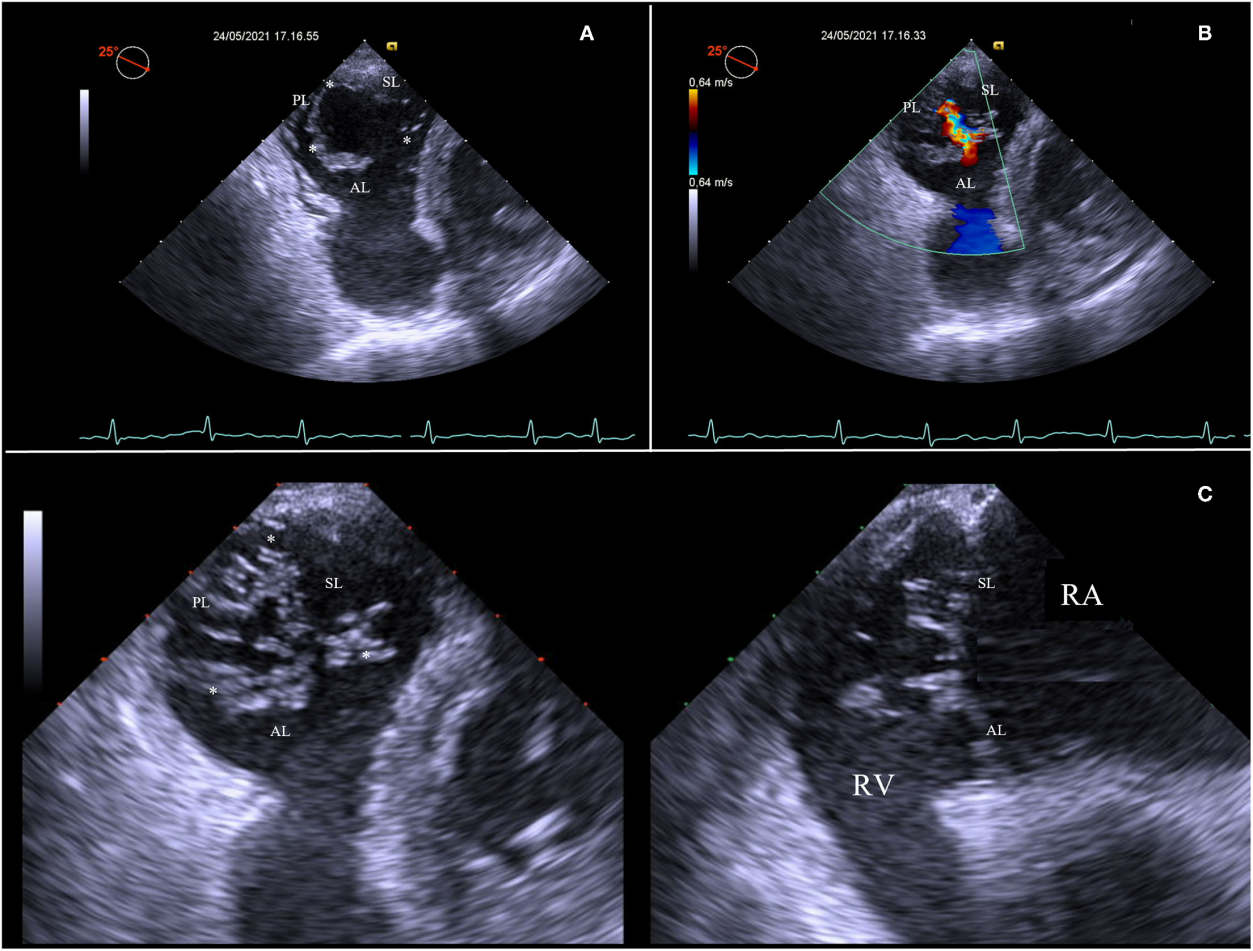
Short-axis transgastric view, identifying all three leaflets and commissures (asterisks) **(A)**, as well as the origin and distribution of the regurgitation jet along all the rim of coaptation **(B)**. Transgastric biplane view focusing on tips of leaflets **(C)**. AL, anterior leaflet; PL, posterior leaflet; RA, right atrium; RV, right ventricle; SL, septal leaflet.

Then, advancing the TEE probe into the stomach results in transgastric views, which enables to obtain optimal view settings to (Figure 2):

- Identify all tricuspid leaflets and commissures. The transgastric short-axis view is the only 2D view that can simultaneously visualize all three TV leaflets;
- Visualize zones of coaptation and all coaptation gaps;
- Analyze the distribution of the TV regurgitation;
- Align the view parallel to the annulus of the TV and focus on the leaflets tips at closure, using the biplane modality.

Three-dimensional echocardiography has greatly improved the accuracy of TV visualization and identification of all anatomical components, eliminating the need for mental reconstruction starting from multiple 2D planes (15). However, since the anterior position of the TV, exacerbated in the dilated RV, 3D imaging may have some limitations. First, it may be tough to visualize the leaflets completely because the TV tissue is thin and rarely thickened. Therefore, an acoustic drop-off may usually be present on 3D reconstructions. In addition, 3D imaging of TEE may be of poorer quality than TTE because the anatomic proximity of the transthoracic transducer to the anterior right heart may facilitate 3D reconstruction. Therefore, it is necessary to obtain multiple volumes from different views to characterize the components of the valve fully. In addition, the surrounding structures should always be included in the volume acquired in order to help to clarify leaflet identification: the aortic valve/aorta for identifying the anterior leaflet, and the interatrial septum/mitral valve for the septal leaflet (6). However, how best to display the valve in 3D remains controversial. Having the septal leaflet as an anatomical reference, there are four classic “en face” atrial perspectives: the anatomical perspective where the septal leaflet is located at 9 o'clock, the bi-caval perspective where the septal leaflet is located at 12 o'clock, the transgastric view where the septal leaflet is located at 3 o'clock, and finally the surgical view where the septal leaflet is situated at 6 o'clock (16).

The 3D approach may also help to improve the accuracy of quantitative Doppler methods and provide integrative criteria for 2D assessment of TR grade. Velayudhan et al. found a 3D vena contracta area (VCA) >0.75 cm^2^ as the most sensitive cutoff value for severe TR (sensitivity 85.2%; specificity 82.1%) (17), whereas Chen et al., found that a 3D VCA of 0.36 cm^2^ was the best cutoff (sensitivity 89%; specificity 84%) (18). This variability can be explained by the different 2D criteria used to define severe TR (17–20). More recently, 3D Proximal Isovelocity Surface Area (PISA) has been used to quantify the Effective Regurgitant Orifice Area (EROA) of TR, with a good correlation with 3D VCA (*r* = 0.97) (21).

Finally, the echocardiographic study aims to assess the structural integrity of the TV leaflets and distinguish functional from primary TR. Moreover, the presence and the spatial relationship with pacemaker leads can be identified, enabling the diagnosis of pacemaker-related regurgitation. The TV area can be measured by planimetry, usually using a 3D approach, since the spatial structure of the TV does not allow a correct alignment in a short axis on the tips of the valve leaflets (22). Other adjunctive criteria for describing the TV geometry are annulus size and leaflet coaptation. Annular size may be quantified in the apical four-chamber or mid-esophageal four-chamber RV focused view, at end-diastole, being 2D measurement underestimated when compared with 3D echocardiography, CMR, and CT scan (23–25). Additionally, the leaflet coaptation can be assessed and quantified in terms of tenting height, tenting area, and tenting volume at end-systole, by 2D and 3D imaging. Severe functional TR is associated with a tenting height of >8 mm and tenting area of >1.6 cm^2^ (26).

Similar to the complex TV echocardiographic assessment, imaging of the right heart is equally difficult due to the unusual shape of the RV and its location within the chest cavity (27). Recommended projections are: standard apical four-chamber, RV-focused apical four-chamber, and modified apical four-chamber view. Speckle-tracking imaging can provide adjunctive information by assessing RV myocardial deformation and evaluating cardiac function and mechanics. Nevertheless, the method is limited by the ability to obtain adequate images for RV strain evaluation, because of the complex shape, geometry, and position of the RV in the chest (28).

## The Role of CT

Cardiac magnetic resonance allows the acquisition of high spatial resolution 3D data of the TV and provides valuable information about the geometrical variations of the tricuspid complex in patients suffering from TR. Multiphasic, cardiac gated image acquisition also enables a complete assessment of biventricular volumes and function (29). The isotropic resolution makes it possible to reconstruct data sets in any desired plane without losing the ability to obtain exact measurements at a later time (30). The image quality for analysis should be optimized using specific CT acquisition protocols focusing on the right-side chambers (31). With a higher number of detectors, the new generation of CT scanners permits shorter breath-hold and lower radiation dose, as well as less contrast medium utilization. Contemporary, CT enables the simultaneous evaluation of coronary, pulmonary, and thoracic conditions (31).

Anatomical changes of TV and quantification of right-side function and remodeling can be assessed using CT imaging, with the aim of understanding the underlying mechanism of functional TR, defining the anatomical suitability for current technologies, and planning TV intervention.

Specifically, CT imaging allows to define:

- dimensions and morphology of the TV annulus
- location of commissures
- tethering height, angle, and area
- anatomical regurgitant orifice area (AROA)
- RA and ventricular dimensions
- anatomic relationships with surrounding structures
- the device landing zone
- prediction of optimal fluoroscopic projections for device implantation
- vascular access route.

**Figure 3:**
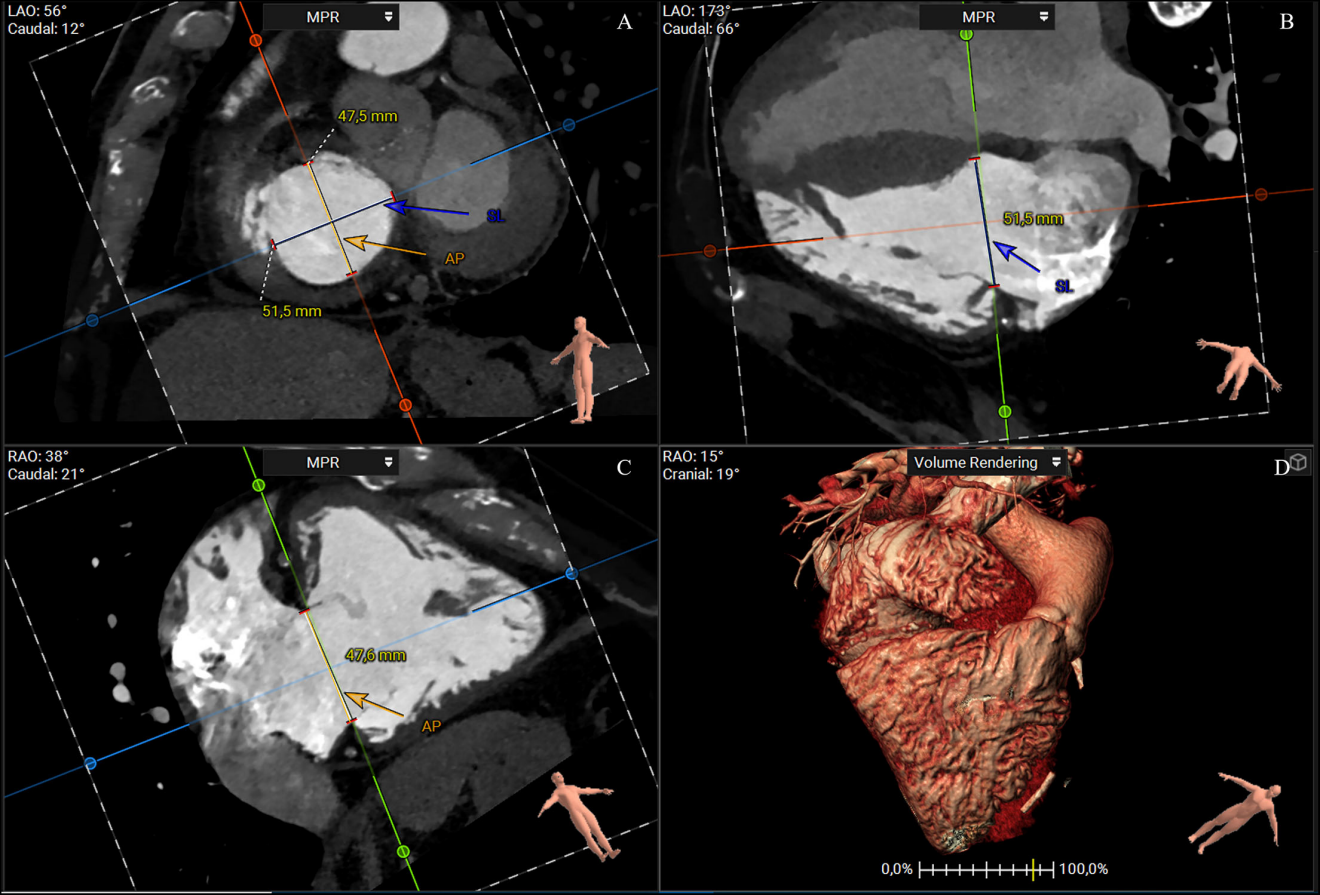
CT multiplanar reconstruction showing the short-axis plane at the annular level **(A)**, the derived four chamber **(B)**, two chamber **(C)**, and volume rendering of the right chambers **(D)**. The annulus septal-lateral diameter is measured in septal to lateral direction (**A**, blue line) and coincides with the annulus measurement in the four-chamber view **(B)**. The anteroposterior diameter is orthogonal at the previous one (**A**, orange), and coincides with the measurement in the two-chamber view **(C)** (3mensio Structural Heart; Pie Medical Imaging, Maastricht, The Netherlands). AP, anteroposterior; SL, septal-lateral.

**Figure 4:**
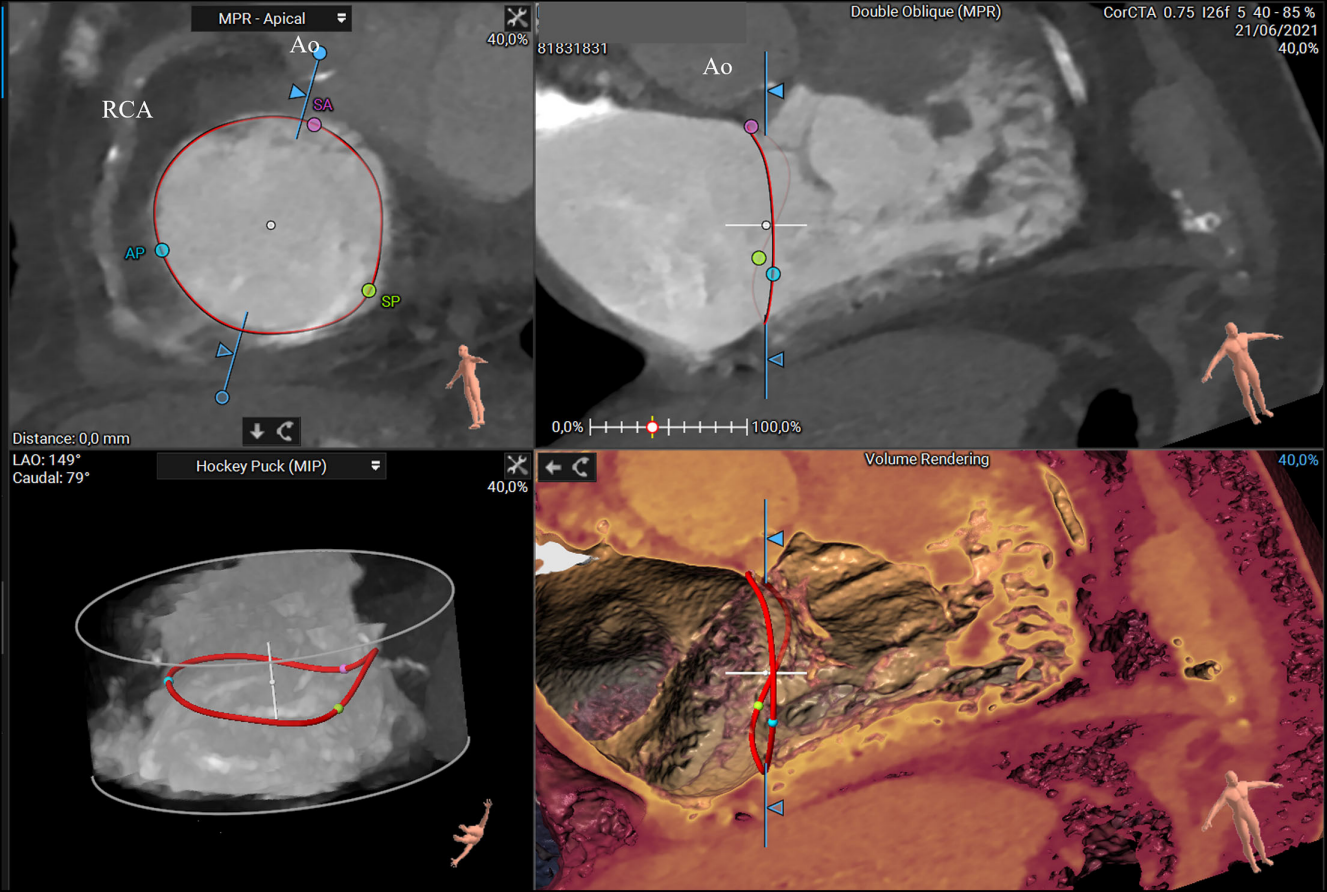
CTassessment of the tricuspid annulus using a semiautomated software-based approach (3mensio Structural Heart; Pie Medical Imaging, Maastricht, The Netherlands). Annulus and commissures are identified in end-systole in 2D and 3D view. The 3D reconstruction of the tricuspid annulus shows a non-planar structure saddle-shaped with two high points (anteroseptal and post-erolateral segments) and two low points (anterolateral and post-eroseptal segments). Ao, aorta; AP, anteroposterior commissure; RCA, right coronary artery; SA, septal-anterior commissure; SP, septal-posterior commissure.

A short-axis plane can be reconstructed on the annular level using multiplanar reconstruction starting from RV two- and four-chamber views. Cross-sectional area, perimeter, septolateral, and AP diameters can be obtained with excellent intra- and interobserver reproducibility (25). The cross-sectional area and perimeter can be assessed by planimetry or using semiautomated software (32–34). The septal–lateral diameter is the maximal distance in septal to the lateral direction and coincides with the annulus measurement in the four-chamber view. The AP diameter is orthogonal to the septal–lateral one. It coincides with the measurement in the two-chamber view with the anterior and posterior wall visualization and the respective leaflets (Figure 3). Because of the dynamic variability in annular size, dimensions should be achieved both in end-systole and mid-diastole. Moreover, since the typical non-planar saddle-shaped structure of the tricuspid annulus, the 2D approach for obtaining measurements does not accurately address the complex annular geometry. Therefore, a dedicated 3D semiautomated software can be valuable in overcoming this disadvantage (Figure 4) (32–34).

Once the annulus has been manually or automated traced, commissures can be located, taking into account their unique anatomical findings. The fan-shaped chords can help in commissural identifications. Precisely, the AS commissure is placed anteriorly, just below the first right coronary tract and the anterior aortic valve cusp. At this site, it is possible to observe a short fan-shaped chorda, arising directly from the septal band of the crista supraventricularis or from a small papillary muscle on that band (8). The PS commissure is placed posteriorly after where the coronary sinus enters the atrium. Anatomically, it has three landmarks: a fan-shaped chorda, a papillary muscle, and a fold in the septal leaflet (8). The AP commissure is placed in correspondence of the free RV wall, usually below the right coronary artery (RCA), roughly at the acute margin. It is well-identified by a fan-shaped chorda and the anterior papillary muscle, which points toward this commissure. Once identified, distances between commissures and distances between the centrum of the TV and commissures can be measured.

In the two- and four-chamber views, it is possible to obtain additional planes parallel to the TV for optimizing the visualization of the tricuspid leaflets. The grade of leaflet tethering, including tenting height, angle, and area can be obtained with a good to excellent intra- and interobserver variability (25). The degree of tethering of the anterior leaflet is measured in the reconstructed two-chamber view, whereas the degree of tethering of the septal and posterior tricuspid leaflets is measured in a reconstructed four-chamber view. The extend of leaflet tethering is always measured in mid-systole.

**Figure 5:**
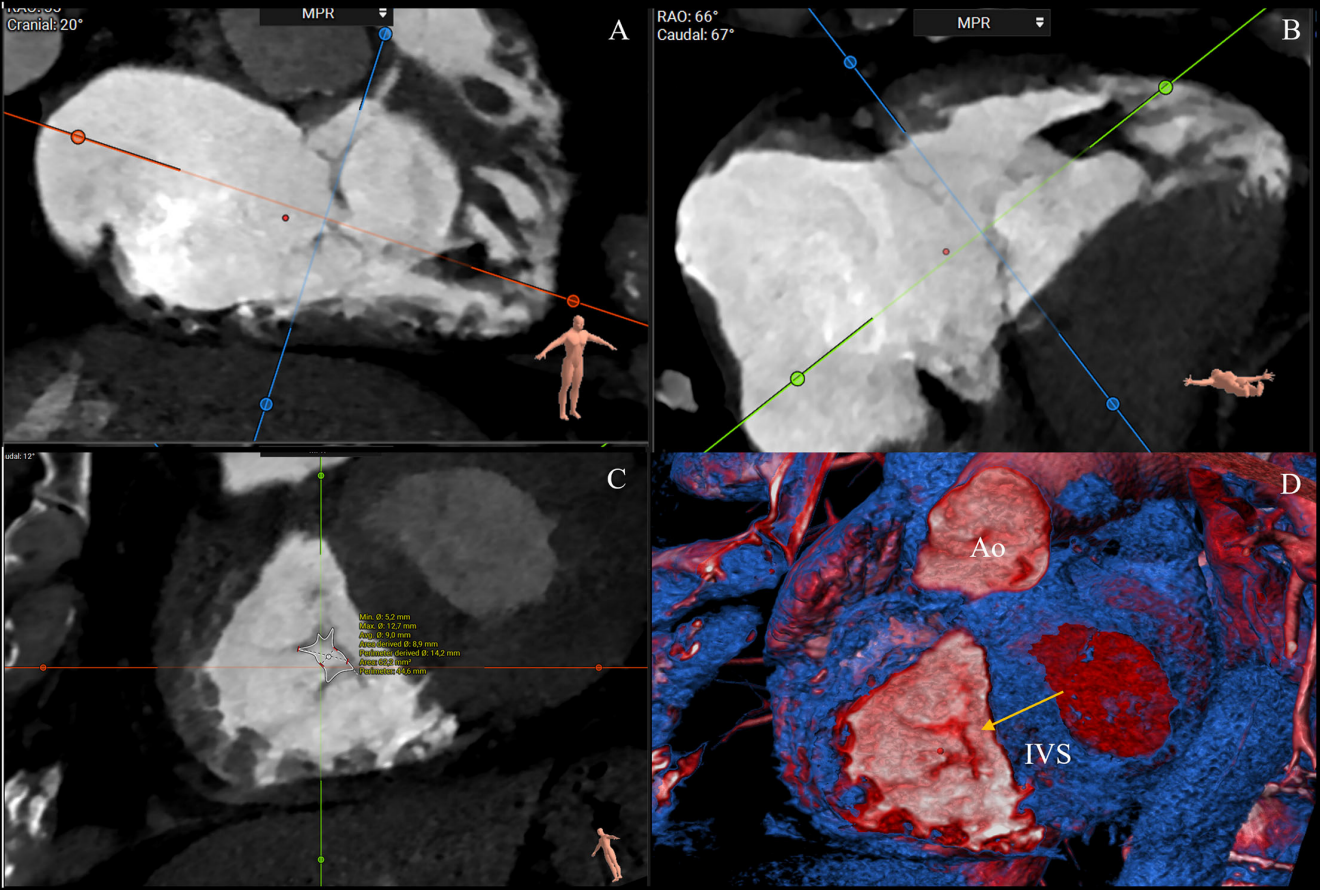
CT assessment of the anatomical regurgitant orifice area (AROA) using a manual approach. Multiplanar reconstruction is performed with the reformation planes aligned with the narrowest portion of the regurgitant orifice during mid-systole **(A,B)**. Then, the contours of the AROA are manually traced on the reconstructed short axis **(C)** and tridimensional visualized in the volume rendering apical view (**D**, arrow) (3mensio Structural Heart; Pie Medical Imaging, Maastricht, The Netherlands). Ao, aorta; IVS, interventricular septum.

Additionally, helpful information obtained by CT analysis is the AROA measurement, which may be used as a potential flow-independent anatomic parameter of TR severity, supplementing and enriching the traditional echocardiographic parameters (35, 36). For this purpose, multiplanar reconstruction is performed with the reformation planes aligned with the narrowest portion of the regurgitant orifice during mid-systole and then, the contours of the AROA are manually traced on the short axis (Figure 5) (35). In a recent study by Lopes et al., the tricuspid AROA measurement resulted reproducible, reliably reflected TR severity assessed by TEE, strongly correlated with 3D VCA, and modestly correlated with right-sided chamber remodeling (36).

Measurements of the right chambers obtained with CT imaging may also be valuable in assessing the anatomic feasibility of a transcatheter therapy and planning the procedure.

In a complete transfemoral approach, the RA must allocate the delivery catheter and have adequate space to permit safely catheter's movements. In case of transcatheter TV replacement, RV volume, as well as the distance between the annular plane and the RV apex and location of the papillary muscles are of interest with regard to the protrusion of the prosthesis into the RV. For quantification of RV volumes and function, semiautomated software can be used.

**Figure 6:**
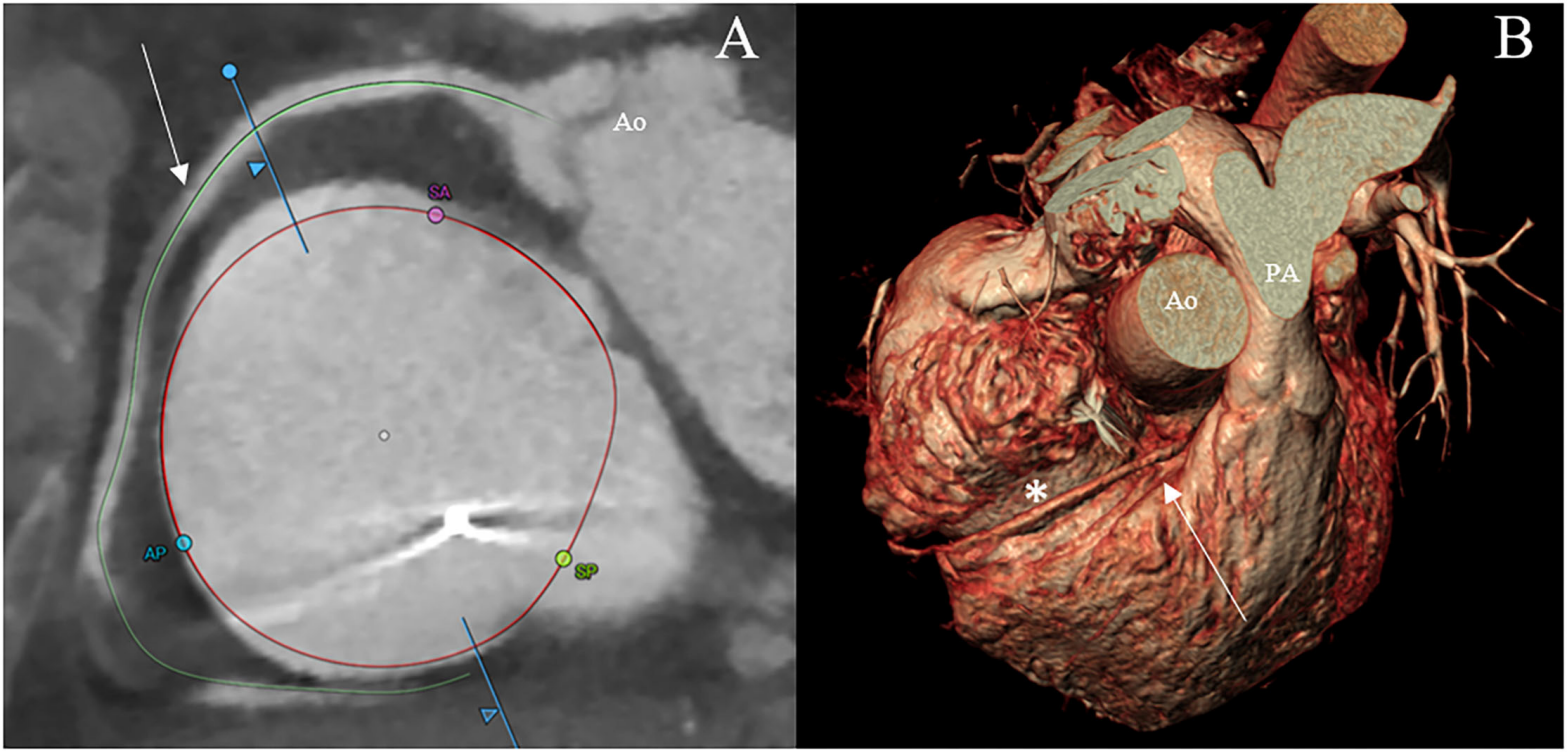
CT scan visualization of the tricuspid annulus (asterisk) and its relationship to the right coronary artery (arrow) using a semiautomated software (3mensio Structural Heart; Pie Medical Imaging, Maastricht, The Netherlands) **(A)** and the volume renderying technique **(B)**. Ao, aorta; AP, anteroposterior commissure; PA, pulmonary artery; SA, septal-anterior commissure; SP, septal-posterior commissure.

Furthermore, assessment of the RCA course and its distance to the annulus, simulation of virtual device implantation, and prediction of an optimal projection angle are part of a comprehensive CT-based pre-procedural assessment (Figure 6) (34).

Finally, the measurements of the inferior vena cava (IVC) along the vessels and at the transition level with the RA, as well as the anatomical relationship of the IVC and the annulus must be examined to plan the procedure. In the case of heterotopic caval valve implantation, additional measurements are obtained in regards to the first hepatic vein. Dedicated semiautomated software can be helpful in IVC analysis.

## The Role of CMR

Cardiac magnetic resonance is particularly well-suited for assessing cardiac morphology and function. It offers an excellent spatial resolution with the advantage of not using ionizing radiation and iodinate contrast agents. On the other hand, limitations in obtaining high-quality imaging and clear interpretation of reconstructions can occur in the presence of cardiac arrhythmias and intracardiac leads, which create significant artifacts.

The RV size and function can be fully assessed using dedicated RV views from multiple planes. Moreover, CMR allows an accurate estimation of TR grade, in terms of regurgitant volume and regurgitant fraction, using direct (by right and left ventricular stroke volume) and indirect methods (by phase-contrast imaging). However, CMR quantification of TR severity is less established than for other regurgitant valves, and there are no specific cutoff values for severe TR (31, 37). Also, the experience of CMR imaging in the setting of TTVI remains limited (31, 37).

Further studies are needed to evaluate the utility of CMR parameters, in defining the timing and predicting the response to TTVI.

## Anatomic Considerations for Interventions

**Figure 7:**
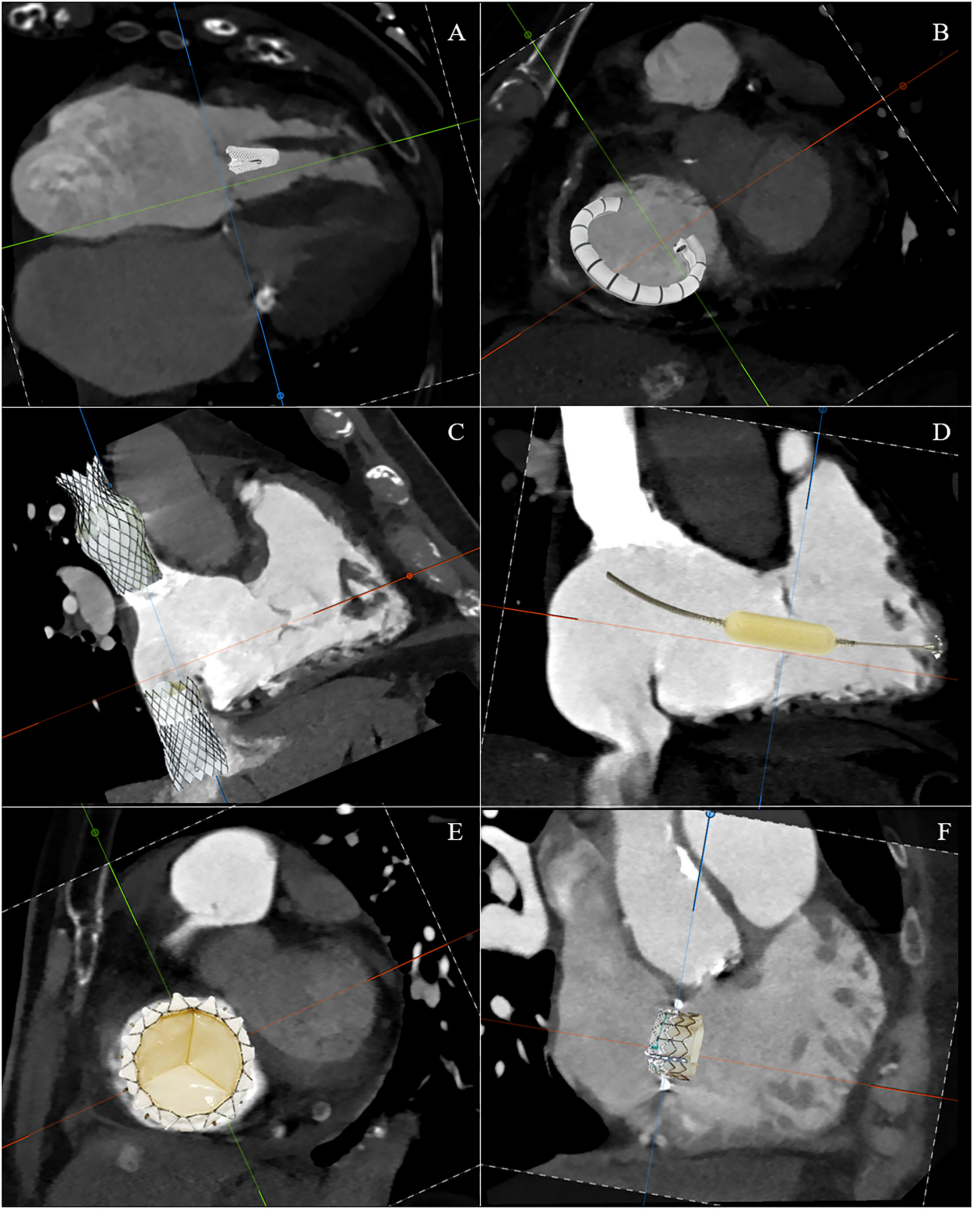
Schematic CT reconstruction of available techniques for transcatheter tricuspid valve interventions: edge-to-edge repair **(A)**, tricuspid annuloplasty **(B)**, caval implants **(C)**, spacer **(D)**, total valve replacement **(E)**, and valve-in-ring replacement **(F)**.

The TTVI can be divided into these five domains: edge-to-edge repair, tricuspid annuloplasty, caval implants, spacer, and total valve replacement (Figure 7). However, there are a number of issues that require a thorough understanding of the morphology of the right side and the process by which secondary TR occurs in order to recognize the role of transcatheter interventions. First, there are multiple access sites possible: vena cavae, direct transatrial, and direct transapical. Second, there are multiple targets of device attaching: annulus, leaflets, vena cavae, and myocardium. Third, the presence of adjacent structures, which on the one hand can be anatomical markers during the procedure, but on the other hand must also be taken into account to avoid complications. Finally, understanding the anatomy and pathophysiology of the disease in an individual patient is the critical point for selecting a specific device for that patient. Table 1 summarizes the anatomic findings and the relative considerations for TTVI.

**Table 1:** Anatomic considerations for transcatheter tricuspid valve intervention (TTVI).

**Anatomic findings**	**Considerations for TTVI**
**Leaflets and commissure**	
- Three leaflets, but variable for deep clefts, scallops and folds. - Very thin leaflets - Larger anterior leaflet with the greatest motion - Posterior leaflet with a variable number of scallops - Short and less mobile septal leaflets - Commissures supported by numerous fan-shaped chords - Commissures usually do not reach the annulus, but several millimeters of leaflet tissue remain sometimes in the form of small scallops	- Imaging leaflet anatomy may be challenging - Thin leaflets are not ideal for anchoring devices - Greater leaflet motion (especially anterior leaflet) may cause high leaflet stress - Maneuvering to capture short and multiscallops leaflet may be difficult - Edge-to-edge devices positioned in commissural region may interfere with the coaptation due to fan-shaped chords distortion.
**Tricuspid annulus**	
- Large valve orifice (normal orifice area of 7–9 cm^2^, increased in TR) - Saddle-shaped/flat structure - Dynamic along the cardiac cycle - Discontinuous fibrous support - Usual absence of calcification	- Stenosis is unlikely with central orifice devices - Stability of devices within the tricuspid annulus may be challenging - Disadvantageous landing zone for prosthesis - Suboptimal anchoring - Risk of paravalvular regurgitation
**Chordae and papillary muscles**	
- The anterior papillary muscle is the largest, supplying chords to the anterior and posterior leaflets - Septal leaflet chords insert directly into the septum or with multiple, small papillary muscles - A large number of chords with various patterns composed of straight collagen bundles, which make chords little distensible	- Papillary muscles serve as an imaging landmark for leaflets and commissures - Marked tethering results from dilation of the right ventricle or displacement of papillary muscles - Catheters and devices may imping with chordae - Location of the moderator band and prominent trabeculae regarding protrusion of the prosthesis into the RV should be assessed
**Surrounding structures**	
- Thin-walled and markedly dilated RA - Dilated RV - SVC: mean length ~7 cm, maximum diameter ~2 cm, funnel-shaped ostium in case of large RA - IVC: extremely dilated and distorted - Coronary sinus enters RA at PS commissure - No continuity between inflow and outflow tract - RCA runs within the AV groove (variable transverse distance from annulus) - AVN, bundle of His crosses the septal leaflet attachment 3–5 mm posterior to the AS commissure - Non-coronary sinus of Valsalva is close to anterior/superior annulus and AS commissure - Pacemaker leads may interfere with leaflet mobility	- Large space to maneuver devices but more difficult for imaging - Adequate distance between the RV apex and the annular plane is mandatory in spacer device and orthotopic prosthesis implantation - Venous access may be limited by SVC diameters and irregular shape. - IVC-annular angle regard the TV plane may pose issues for device placement - Anatomic landmarks (coronary sinus, non-coronary aortic sinus, RCA) - Little risk for outflow tract obstruction - Risk of RCA injury - Risk for heart block with devices in AVN region - Intracardiac leads can create significant artifacts in CT and CMR imaging study, compromising image quality and interpretation - Intracardiac leads can interfere with device implantation

*AVN, atrioventricular node; AS, anteroseptal; CMR, cardiac magnetic resonance; IVC, inferior vena cava; PS, post-eroseptal; RA, right atrium; RCA, right coronary artery; RV, right ventricle; SVC, superior vena cava; TR, tricuspid regurgitation; TV, tricuspid valve*.

The choice of the most suitable transcatheter therapeutic option in a single patient remains challenging (38, 39). This is because the implantation of a device within the tricuspid annulus is challenged by the variable anatomy of the TV, very thin leaflets, and the anatomic proximity to other structures (coronary sinus, His bundle, atrioventricular node, and RCA). Furthermore, the 3D geometrical shape of the tricuspid annulus is non-planar, often severely dilated and dynamic over the cardiac cycle. In addition, the TV in the setting of functional TR, usually does not show calcifications that could facilitate the anchoring of transcatheter prostheses.

Consequently, a multimodality cardiac imaging focusing on the anatomy and function of TV apparatus, right-side chambers, and adjacent structures is mandatory for diagnosis and prognosis and the planning of transcatheter therapy.

Indeed, specific imaging techniques for each transcatheter procedure are required to assess anatomic feasibility and select the correct procedure for an individual patient (Table 2).

**Table 2:** Transcatheter tricuspid valve interventions: imaging needs and anatomical feasibility.

**Type of intervention**	**Target structure**	**Imaging need**	**Favorable anatomical conditions**	**Unfavorable anatomical conditions**
Edge-to-edge	Tricuspid leaflets	- Location of the largest vena contracta (echo) - Coaptation, length tethering and motion of the leaflets (echo)	- Small coaptation gap (<7 mm) - AS or PS jet location - Large annulus - Small RV dimensions	- Diffusely degenerated leaflets - Large coaptation gap (>7–10 mm) - Severe tethering (depth > 10 mm) - Pacemaker lead with leaflet tethering - Poor echo windows
Tricuspid annuloplasty	Tricuspid annulus	- Annulus dimensions (CT) - Course of RCA along the AV groove (CT) - Distance between RCA and annulus (CT)	- Annular dilatation mainly due to RA dilatation - Variable leaflets anatomy (multiscallops, clefts) - Small RV dimensions - Favorable RCA course	- Large coaptation gap (>10 mm) - Very large annulus - Lead induced TR - Poor echo windows
Caval implants	Superior and inferior caval veins	- Dimensions of caval veins (CT) - Distance between the cavoatrial junction and the first hepatic vein (CT) - Dimension of RA (CT) - RV systolic function (echo-CMR)	- Palliative care (not favorable anatomy) - Pacemaker leads - Severe RV dilatation	- Severe RV dysfunction - Severe IVC dilatation - Inadequate hepatic vein anatomy - Contraindication for OAC
Spacer system	Tricuspid leaflets	- Location of the largest vena contracta (echo) - Annulus dimensions (echo-CT) - RV dimensions (echo-CT) - Distance from the tricuspid annulus plane to RV septal free wall (CT) - Location of the RV anchoring target (RV) - Dimensions of the left subclavian and axillary veins (CT)	- Small coaptation gap (<7 mm) - Large annulus	- Unfavorable anatomy of RV - Subclavian and axillary veins occlusion - Pacemaker lead - No functional etiology
Valve replacement	Tricuspid valve	- Dimensions and morphology of the TV annulus (CT) - RA and RV dimensions (echo-CT) - Distance between the RV apex and the annular plane - Location of moderator band, prominent trabeculae and papillary muscle (CT) - Course of RCA and distance between RCA and annulus (CT) - Identification of the AVN region and aortic Valsalva sinus (CT) - Course and dimension of caval veins and angle between caval veins and TV plane (CT) - Prediction of fluoroscopic angulations (CT) - For ViV/ring, mechanism of failure (Echo, CT) - For ViV/ring, dimensions of the ring/valve (CT)	- Large coaptation gap (>7–10 mm) Severe tethering (depth >10 mm) - Poor echo windows - Prior surgical tricuspid therapy - Independent of TV morphology (rheumatic, carcinoid, multiscallops, cleft) - Future ViV possible	- Small RV dimensions - Very large annulus - Pacemaker lead

*AS, anteroseptal; AV, atrioventricular; CMR, cardiac magnetic resonance; OAC, oral anticoagulation therapy; PS, posteroseptal; RA, right atrium; RCA, right coronary artery; RV, right ventricle; ViV, valve-in-valve*.

## Edge-to-Edge Repair

Edge-to-edge repair devices act on tricuspid leaflets with the aim to improve the coaptation in the regurgitant valve. Currently available devices include the Triclip system (Abbott Vascular, Santa Clara, CA, USA), which grasps and coaptates leaflets (40), and the Pascal device (Edwards Lifesciences, Irvine, CA, USA), which combines independent leaflet grasping with the presence of a central spacer that increases coaptation by occupying the regurgitation orifice (41).

During the pre-procedural examination for edge-to-edge repair, a complete assessment of the TV anatomy is mandatory, including where the largest coaptation gap is located and which leaflets are more tethered. The echocardiographic 3D en-face view of the TV, the transgastric short axis, and the mid/deep esophageal RV inflow/outflow with simultaneous biplane view permit accurate assessment of the leaflets and commissures, as well as the largest coaptation gap. Preoperative CT imaging is not routinely performed for screening patients with TR who are candidates for edge-to-edge valve repair (34).

Data from published experiences demonstrate that the best results occur by attaching the anterior and/or posterior leaflet to the septal leaflet, which can also reduce annular dimensions without distorting the valve (42). This zone is also the simpler to approach due to the more favorable angle between IVC and TV plane (43). Moreover, in experimental models of functional TR, clipping the septal and anterior leaflets was associated with a significant reduction in TR grade and a considerable increase in cardiac output, particularly when the devices were placed centrally instead of at commissural level (44). The device positioned at the commissural level may distort the coaptation resulting in a worse result.

The favorable and unfavorable anatomical conditions for the edge-to-edge repair are listed in Table 2.

## Tricuspid Annuloplasty

Annuloplasty devices aim to reduce the annular size in functional TR using a transfemoral ring- or suture-based approach. Precisely, the Cardioband (Edwards Lifesciences) consists of a flexible ring with multiple anchors attached to the annulus (45). The TriAlign (Mitralign Inc., Teksbury, MA, USA) consists of a suture-based transjugular device with two pledgets placed at the PS and the AP commissures, which are then cinched to obliterate the posterior tricuspid leaflet, resulting in a bicuspidalized TV and reduction of annular dimensions (46). The Tricinch device (4Tech Cardio, Galway, Ireland), currently no longer available for clinical use, consists of a nitinol coil for annular fixation positioned in the pericardial space, a connecting tension band, and a stent in the IVC that maintains the desired tension to achieve annular remodeling (47).

Detailed measurements of the tricuspid annulus are obtained by CT images. But the major concern related to annuloplasty devices is the risk of RCA injury as it runs closely and parallel to the tricuspid annulus. Therefore, it is imperative to routinely assess the course of the RCA and the distance between the vessel and the annulus by CT reconstruction. In addition, valve tethering should be assessed with both CT and echocardiography.

The favorable and unfavorable anatomical conditions for the annuloplasty are listed in Table 2.

## Caval Implants

Heterotopic implantation of valves in the caval veins aims to reduce blood backflow and prevent systemic venous congestion. The technique counts balloon-expandable transcatheter aortic valve prostheses (Sapien XT, Edwards Lifesciences) implanted in the inferior (and superior) caval veins (48); the TricValve system (P&F Products and Features, Vienna, Austria), which consists of two dedicated self-expanding valves, for the superior and inferior caval veins (49); and the customized Tricento device (NVT AG, Muri, Switzerland), which is deployed from the superior to the IVC (50).

The most important parameters to consider in cardiac imaging for heterotopic implants include:

- Systolic backflow reversal in the inferior caval vein on echocardiography.
- Accurate measurement of the diameters of caval veins using CT. The IVC is measured at the level of the cavoatrial junction and at the level of the first hepatic vein. In addition, the distance between the cavoatrial junction and the most superior hepatic vein is measured to avoid obstruction of this vein during valve implantation. The superior vena cava is measured at the cavoatrial junction. In case of large RA, the cavoatrial junction may have a funnel shape, increasing the valve migration risk.
- Measurement of the RA is required for the Tricento device.
- Assess whether RV function is preserved because RA pressure increases and increased RV and atrial volume overload persist after the procedure, leading to further adverse remodeling and RV failure and preventing symptomatic benefit.

The favorable and unfavorable anatomical conditions for the caval implant are listed in Table 2.

## Spacer

The Forma spacer device (Edwards Lifesciences) aims to minimize the leaflet coaptation gap caused by tricuspid annular dilatation and leaflet tethering, through a foam-filled polymer balloon (spacer) deployed at the level of the TV and an anchoring system placed in the RV septal free wall groove (51, 52).

The pre-procedural imaging checklist includes:

- Measurement of the largest vena contracta or coaptation gap by echocardiography
- Measurement of the TV annulus, preferably from CT
- Measurement of RV dimensions and distance between the annulus plane and RV septal free wall groove, using CT or CMR
- CT imaging of the proposed anchor site for possible obstruction to the passage of the rail and anchor system
- Dimensions of the left subclavian and axillary veins, which will be used to introduce the system. The CT is recommended to assess these measurements.

The favorable and unfavorable anatomical conditions for the space technique are listed in Table 2.

## Valve Replacement

Transcatheter TV replacement is a promising therapeutic tool that is still in its infancy. However, the procedure presents significant technical challenges due to the anatomical peculiarities of the TV annulus, which is enlarged, dynamic during the cardiac cycle, and without calcifications in the landing zone for the prosthesis. This can result in incomplete apposition of the prosthesis in the native annulus with consequent possible significant paravalvular regurgitation. Moreover, the anatomical relationship with surrounding cardiac structures (i.e. aortic valve, RCA and atrioventricular node in the triangle of Koch) poses the risk of potential procedural complications. The NAVIGATE (NaviGate Cardiac Structures, CA, USA) is a self-expanding bioprosthesis for orthotopic replacement implanted through a transjugular or transatrial 42-Fr approach and consists of three xenogeneic pericardial leaflets, seated in a tapered nitinol stent with atrial winglets and ventricular graspers to anchor the tricuspid annulus and leaflets without protruding into the adjacent chambers. However, the short series published so far have shown the feasibility and safety of this procedure (53, 54). Recently, the first 28 Fr transfemoral implantation of the EVOQUE TV Replacement System (Edwards Lifesciences) was reported, and a clinical trial is underway (TRISCEND Study-NCT04221490) (55). Other devices such as TriSol (TriSol Medical), Lux (Jenscare Biotechnology), and Topaz (TRiCares SAS, Paris, France) have been tested but have yet to be used in the clinical setting (42, 56, 57).

Specific CT measurement protocols are needed for pre-procedural planning, including a comprehensive assessment of valvular and subvalvular anatomy, prosthesis sizing, vascular access route, and prediction of fluoroscopic angles. This study can be additionally supported by virtual prosthesis placement to predict implantation depth, prosthesis selection, and anchoring possibilities.

Specifically, the pre-procedural imaging checklist should include (34):

- dimensions and morphology of the TV annulus
- RA and ventricular dimensions
- distance between the RV apex and the annular plane and location of the moderator band and prominent trabeculae with regard to the protrusion of the prosthesis into the RV during implantation
- anatomic relationships with surrounding structures
- analysis of vascular access route and relationship between caval veins and TV plane.

On the other hand, in case of transcatheter TV-in-valve and valve-in-ring procedures, the measurements should be routinely assessed by a dedicated CT analysis rather than the nominal size documented in the surgical report.

The favorable and unfavorable anatomical conditions for the orthotopic valve replacement are listed in Table 2.

## Conclusions

With the ongoing rise in transcatheter TV interventions, procedural success extremely depends on precise and comprehensive pre- and periprocedural imaging. Multimodality imaging, including 2D and 3D echocardiography, as well as CT, is crucial in the complete anatomic evaluation of the right heart and TV, which are the target for current TTVI. On the other hand, the rapid growth and promising success of these procedures have been accompanied by advances in imaging techniques that have helped to accurately visualize the TV and its anatomic relationships, allowing appropriate selection of patients for the various techniques of transcatheter tricuspid repair.

## Author Contributions

VC contributed with design and drafting of the manuscript. MC, MB, and SM contributed with revision of the content. GU contributed with conception, revision, and final approvement of the manuscript. FG contributed with conception of the paper. All authors agree to be accountable for the content of the work.

## Conflict of Interest

The authors declare that the research was conducted in the absence of any commercial or financial relationships that could be construed as a potential conflict of interest.

## Publisher's Note

All claims expressed in this article are solely those of the authors and do not necessarily represent those of their affiliated organizations, or those of the publisher, the editors and the reviewers. Any product that may be evaluated in this article, or claim that may be made by its manufacturer, is not guaranteed or endorsed by the publisher.
